# Developing Forest Therapy Programmes Based on the Health Benefits of Terpenes in Dominant Tree Species in Tara National Park (Serbia)

**DOI:** 10.3390/ijerph19095504

**Published:** 2022-05-01

**Authors:** Martina Zorić, Jelena Farkić, Marko Kebert, Emina Mladenović, Dragić Karaklić, Gorana Isailović, Saša Orlović

**Affiliations:** 1 Institute of Lowland Forestry and Environment, University of Novi Sad, Antona Čehova 13d, 21000 Novi Sad, Serbia; kebertm@uns.ac.rs (M.K.); sasao@uns.ac.rs (S.O.); 2 Academy of Applied Studies, Bulevar Zorana Djindjica 152a, 11000 Belgrade, Serbia; jfarkic@gmail.com; 3 Department of Fruit Growing, Viticulture, Horticulture and Landscape Architecture, Faculty of Agriculture, University of Novi Sad, Trg Dositeja Obradovića 8, 21000 Novi Sad, Serbia; eminam@polj.uns.ac.rs; 4 Public Enterprise ‘Tara National Park’, Milenka Topalovića 3, 31250 Bajina Bašta, Serbia; office@nptara.rs; 5 Forest Therapy Southeastern Europe, Gospodar Jevremova 25, 11000 Belgrade, Serbia; isailovic.gorana@gmail.com

**Keywords:** BVOCs, *Picea abies*, *Abies alba*, Shinrin-Yoku, human health, hiking trails, tourism

## Abstract

Numerous medical studies have shown the positive effects of forests on different aspects of human health. This study deals with the content of major terpenes in dominant coniferous species in Tara National Park, Serbia, in order to explore the potential for the development of a novel health tourism programme based on forest therapy. Main terpenes were analysed using a headspace-sampling technique coupled with gas-chromatography-mass spectrometry (Head-space-GC/MS). Needles of fir and spruce growing in the vicinity of hiking trails were investigated for possibilities to perform such therapy. Major detected terpenes were α-cadinol and spathulenol previously described as antiviral, antitumor, antimicrobial and immunomodulatory agents. The results of the study were favourable and worked well with the existing walking infrastructure in the observed area of the Tara Mountain, as they act as invaluable resources for designing the structured forest bathing walks. The study not only adds to the knowledge in the environmental and public health realm but also to tourism and sustainability studies.

## 1. Introduction

The positive effects of forest ecosystems on human health have been proven by numerous scientific studies across diverse disciplinary fields [1,2,3,4,5]. The necessity for people to more frequently utilise green spaces for health and wellbeing purposes has been brought into even greater focus during the ongoing Coronavirus pandemic [6] Environmentalists, public health professionals and tourism planners alike have recognised that there is an increased need to promote more sustainable approaches to enhancing human wellbeing and improving their immune response through encouraging more frequent utilisation of forest environments [7,8,9]. One such concept to which we pay particular attention in this study is forest therapy, which has become not only a health and wellness trend in recent years but also a much-desired activity in nature-based tourism destinations [9,10].

Forest ecosystems have been long suggested as a non-pharmacological potential health medium. To date, great efforts have been made in the realm of public health to explore the positive effects that spending time in arboreal environments have on human health, generating a large corpus of data to draw from [7]. Researchers have paid increased attention to the effects of forests on physiological relaxation and immune recovery, with Japan being the world leader in forest medicine research [11]. While the positive effects of forests on different health issues, such as high blood pressure, respiratory problems, stress, anxiety and other mental health disorders have been scientifically evidenced, the exact forest factors that are responsible for these positive changes have not yet been explored in more detail [8].

It is claimed that biogenic volatile organic compounds (BVOCs) in the forest air are one of the main forest attributes contributing to human health improvement [12]. Air BVOCs include, among others, the atmospheric trace gas compounds named terpenes, which have been demonstrated to have different biological activities that could contribute to human health. Some of them act as anti-inflammatory, antitumor, antidepressant, antimicrobial, antiviral and sedative agents [13,14,15,16,17,18,19,20,21,22,23,24]. Terpenes are the largest class of plant secondary metabolites and still remain insufficiently identified with about 1000 new structures being added every year [25]. These compounds, when emitted in the air, are highly reactive, leading to their short lifespan which ranges from several minutes to several hours [25]. Building on previous research into this matter, there is a further need to examine the potential of the most common tree species in the examined areas to emit different BVOCs and how they contribute or could be used as a benefit in developing a forest-based therapy programme for preventive actions for public health and tourism purposes. 

The biogenic volatile organic compounds (BVOCs) are plants’ secondary metabolites; the compounds plants use to protect themselves from stress, herbivores and pathogens, but also to communicate with other plants [26]. The significance of these compounds was first researched by atmospheric chemists who were eager to investigate the role of these compounds in air composition [7]. Nowadays this research continues, especially regarding ozone formation and the impact of BVOCs, in general, on air quality in urban areas [27,28]. On the other hand, the role of these compounds is highly important in the pharmaceutical and cosmetical and even the food industry [29]. Most of the investigated roles of these compounds are directly or indirectly connected to human health. 

When emitted into the air, BVOCs, including terpenes, act as highly reactive chemicals, and their lifespan is greatly reliant on the environmental circumstances that are unique to each time and space [25,30,31]. Although the content of BVOCs does not always reflect their emission, the headspace-sampling technique can be used as a tool to gain insight into the emission rate of BVOCs released from plants [32]. 

The goal of this study is to provide more information, and insight for future research involving medical professionals and measuring BVOCs in the air during actual therapy in order to reach more reliable conclusions about the health effects of BVOCs from specific species and locations in strictly defined time and space as well as environmental conditions.

This exploratory study, therefore, aims to identify the main terpenes of dominant coniferous tree species on the mountain Tara: fir (*Abies alba*) and spruce (*Picea abies*). Moreover, it will evaluate the suitability of the existing hiking trails in this area for forest therapy based on the results of the terpenes analysis as well as other relevant environmental, health and tourism factors of the area. 

## 2. Materials and Methods

### 2.1. Study Area

**Figure 1:**
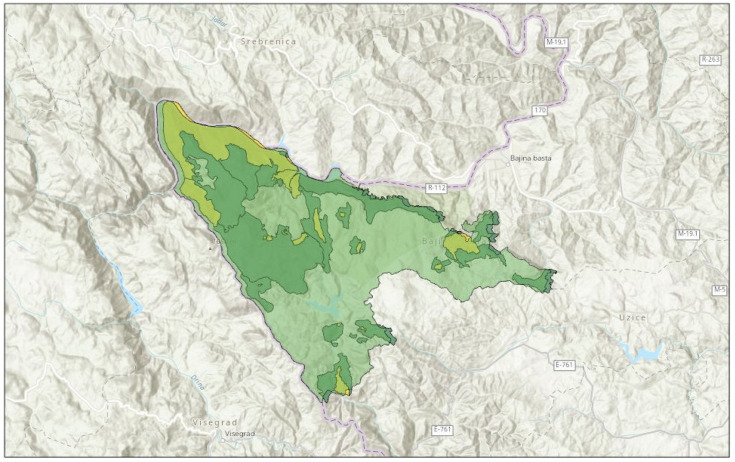
Boundaries of Tara National Park and different levels of protection. In light green- boundaries of Tara National Park; In yellow—first level of protection; In dark green—second level of protection.

Tara National Park is placed in the western part of the Republic of Serbia (Europe) within the territory of Bajina Bašta Municipality. This area encompasses the Tara Mountain, Drina River Canyon, Zvezda Mountain, and Lake Perućac and is under different levels of protection (Figure 1). Tara Mountain is categorized as a medium-high mountain with the highest peak Koziji rid-1591 m and has an average altitude of 1200 m. The management of the natural capital of this area is under the jurisdiction of the Public Enterprise “Tara National Park”. The Park itself covers an area of 24,991.82 ha out of which 13,589.54 ha (54.3%) are forests in the state ownership [33]. Overall, the forest coverage of NP Tara is around 60%, out of which the most dominant (85%) are mixed stands of beech and fir with spruce (*Piceo-Abieti-Fagetum* Čoli. 65) [33].

Tara National Park (NP Tara) is the geographic location within which this study is situated. It is known for its pristine nature, relatively underdeveloped tourism and its scarce infrastructure, which escapes mass tourism and visitor pressures on the mountain. We thus recognised the potential of this area to offer a fertile ground for the development of a new health and wellbeing tourism offer, which will be sustainable for the natural environment, local population and tourists. 

**Figure 2:**
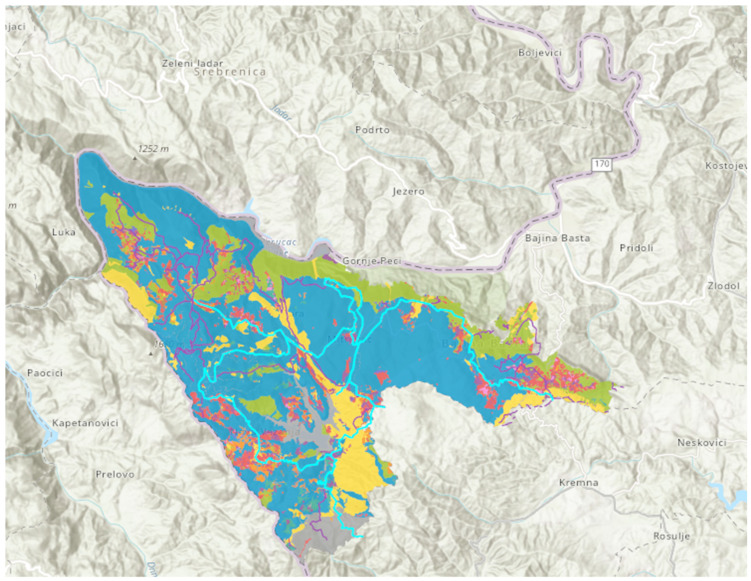
Investigated hiking trails (marked with light blue) in the area of mixed stands of beech and fir with spruce (*Piceo-Abieti-Fagetum*) marked with blue.

Mixed stands of beech (*Fagus sylvatica* L.) and fir (*Abies alba* Mill.) with spruce (*Picea abies* L. Karst) are the most common in the examined area. In order to include the relevance of different tree species and terpenes in this evaluation, we focused on the area encompassing the hiking trails which extends through the mixed forests (*Piceo-Abieti-Fagetum*) (Figure 2). 

### 2.2. Hiking Trails in NP Tara

**Figure 3:**
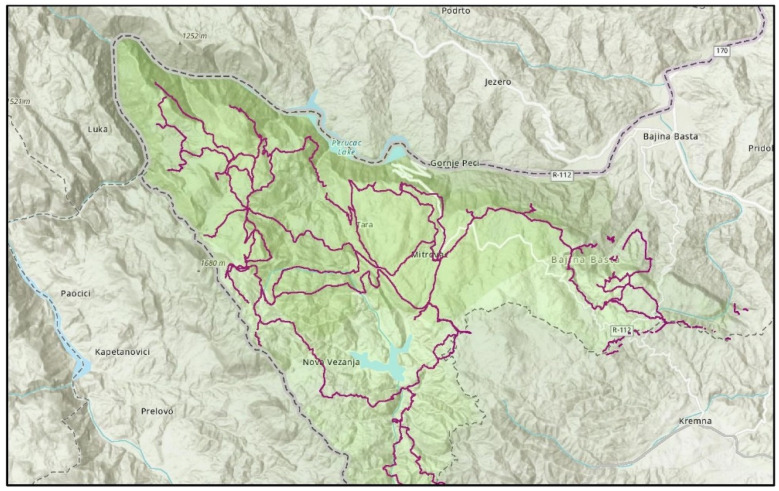
Hiking trails within Tara National Park, marked with purple lines.

There is 290 km of 40 marked hiking trails in Tara National Park, including Viadinarica and the European hiking corridor which are connected to neighbouring and other European countries (Figure 3). All hiking trails differ in multiple aspects, such as distance, elevation, hike duration, and dominant tree species.

The profile of hiking trails represents the combination of the following parameters:

- Hiking route length (km)
- Hiking route elevation (m)
- Hiking route duration (hours)
- Conditional and technical difficulty (ranged from 1 to 10, 1 as no difficulty to 10 as highly difficult)
- Accessibility (if the hiking trail is partially on or near the local road for the security of the users).

All data considered the metric specifications (Table 1) of the investigated hiking trails (length, elevation, and duration) used for the evaluation of specific hiking trails within this study, are publicly available on the website of Tara National Park [34]. 

### 2.3. Plant Material and Terpenes Analysis

For this research, the composition of terpenes in the needles of two common coniferous species, fir (*Abies alba*) and spruce (*Picea abies*) growing in NP Tara was investigated in order to define their potential to positively impact human health. Further, the composition of terpenes, with the focus on monoterpenes and sesquiterpenes, was further investigated since there is a general opinion that these trees’ compounds are one of the main factors that contribute to the improvement of human health [12].

**Figure 4:**
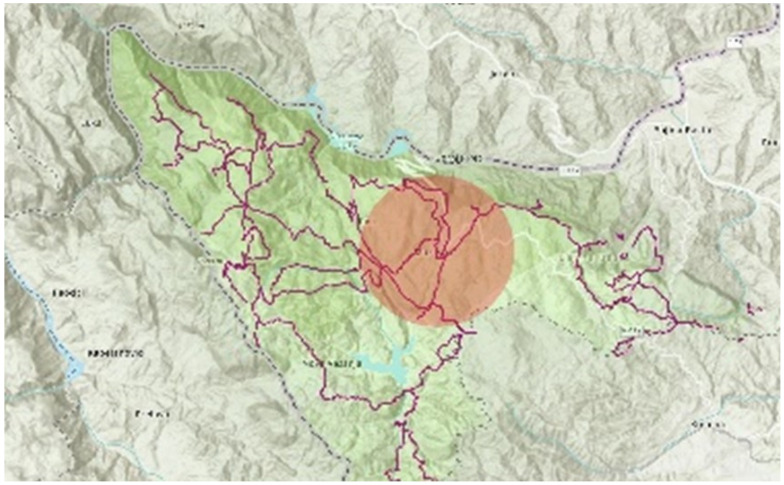
The area of sampling, wider location of tourism centre Mitrovac, marked with red.

The plant material was collected in July 2019. The needles were collected from three individuals of each species, growing in the mixed stands of beech and fir with spruce in the proximity of the investigated hiking trails, and the main tourism centre, Mitrovac (Figure 4). These individuals were fully formed, and medium-aged with no signs of mechanical injuries and/or pathogens. To ensure the examination of terpenes content in the whole canopy of an individual, the samples were taken from the top, the middle and bottom parts of the canopy. The samples fully exposed to sunlight were taken on a sunny day, with no precipitation, around noon, as previously described in our previous research [35].

The composition of terpenes was investigated using a headspace-sampling technique coupled with gas-chromatography-mass spectrometry (Head-space-GC/MS, 7890A, 5975C MSD, Agilent technologies). Before the analysis, dried samples were homogenized and powdered with liquid nitrogen and sealed/crimped into the vials (20 mL) each containing 0.06 g of the investigated material. SPME needle with exposed fibre (SPME Fiber Assembly Carbonex/PDMS, 75 μm, Agilent technologies) was inserted into the sealed vials which were heated at 60 °C for 30 min at the thermal block. After the exposure of the adsorbent, the SPME needle was injected directly into the inlet of the gas chromatograph in the splitless mode. The analytical separation on the GC column was performed for 30 min, with starting oven temperature of 45 °C for 2 min rising to 280 °C for 10 min. The identification of terpenes was performed by using the National Institute of Standards and Technology (NIST) library based on mass spectrums and retention times (RT). The semiquantitative method was used for quantification, and the abundance of each compound was calculated as a percentage of separate peak to total ion chromatogram peak area. For the purposes of this research within this chemical profiling of selected species, the composition and abundance of detected terpenes were observed as the potential of each species and examined the area for use in forest therapy. Mean values of all sample results within investigated species were used to extract major terpenes in fir and spruce among other detected compounds and were afterward discussed.

## 3. Results

### Terpenes Composition in Dominant Species of the Area

**Figure 5:**
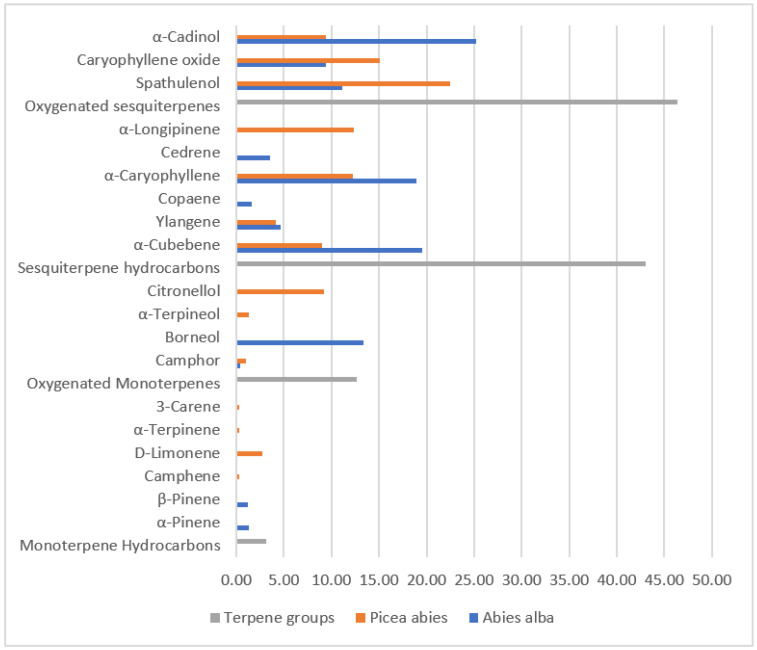
Major terpenes in the examined species based on the mean values (%) of the obtained results.

The analysis of terpenes content in the needles of two dominant coniferous species of the examined area, spruce (*Picea abies*) and fir (*Abies alba*), showed the presence of 12 major compounds (Figure 5). The values (%) of detected compounds varied from 0.28% up to 25.18%, out of which oxygenated sesquiterpenes were the most abundant, followed by sesquiterpene hydrocarbons. In less amount, oxygenated monoterpenes were present, while the least present were monoterpene hydrocarbons. 

The most abundant terpenes were α-cadinol in fir and spathulenol in spruce. 

The phytochemical screening of spruce showed that the most abundant among major detected terpenes were sesquiterpenes: α-cadinol (25.18) and α-cubebene (19.55%) (Table 2). Additionally, in fir samples, sesquiterpenes were dominant as the most abundant were spathulenol (22.44%) and caryophyllene oxide (15.12%). Other detected major sesquiterpenes in needles of the examined species were: α-caryophyllene, yanglene, copaene, cedrene and α-longipinene, with only α-caryophyllene present in both, fir and spruce in concentrations of 18.92% and 12.23%, respectively. 

Considering monoterpenes, the most abundant was borneol, detected in fir (13.40%), followed by citronellol in spruce (9.22%). Camphor was detected in both fir (0.40%) and spruce (1.03%), while camphene, D-limonene, α-terpinene, 3-carene and α-terpineol were only detected in spruce. Other than that, in fir samples, α-pinene and β-pinene were also detected at values of 1.27% and 1.22%, respectively. 

## 4. Discussion

### 4.1. Health Benefits of Terpenes 

The actual impact of terpenes on human health in forest environments is under constant discussion in terms of determining their exact influence, while excluding other forest factors. The main raised questions are concerning the exact concentrations and blends of these compounds in the air that could have an impact on human health. The main reason for this is the high dependability of terpenes on the environmental factors that influence the emission from plants, but also the concentration of these compounds in the air [36]. Different factors: internal (genetic and biochemical) as well as external (abiotic-temperature, light, water availability, wind, ozone, and biotic-animal, plant, and microorganism interactions) control the emission rates of different BVOCs by altering their synthesis, and vapour pressure of diffusion to the atmosphere [37]. Previous studies that focused on measuring the concentration of the BVOCs in the forest air did not focus on the individual compounds, but instead on the dependability of overall BVOCs on the environmental conditions and confirmed its great dependability on temperature and solar radiation [38]. Additionally, previous intentions to create models of emission of BVOCs from different plants species, while not distinguishing individual terpenes, also observed light and temperature as major environmental variables to influence BVOCs emission, but claimed that other factors, although not noted in their research, such as insect or wind disturbance, water stress, etc., could have a significant influence on the BVOCs emissions [39].

Although there is a difference between emitted and stored BVOCs [40], in the present research, we focused on the examination of the content of terpenes in tree species and the content of BVOCs is observed as their potential to emit these compounds in the air, instead of analysing BVOC concentrations in the air samples, since it cannot be constant, and it differs in different seasons and part of the day [38]. This was confirmed in previous research claiming that the lifetime of monoterpenes in the air can last up to 3 h, while sesquiterpenes have a very short lifetime of less than 4 min [25]. Due to the small possibility of reproducibility of the results considering measuring BVOCs in the air, in order not to provide inaccurate information for further medical research, we have used the headspace-sampling technique coupled with the gas-chromatography-mass spectrometry (Head-space-GC/MS) to analyse terpene content in selected species, which are already known to be high BVOCs emitting plants [41,42]. Our goal was to provide an insight on what are the possibilities of dominant tree species in the investigated location in terms of emitting specific BVOCs in the air. This technique is traditionally used to gain insight into the emission blend of a plant, and it can also be a tool to study the emission of BVOCs released by vegetation and compared to other techniques, it better reproduces the genuine scent that could be perceived from the fresh plant [32]. Our goal was to provide possibilities and certain guidelines for further specific medical research since previous shows that even the mild interaction with these compounds in the forest air act beneficial on human health [7]. 

Terpenes and BVOCs in general as bioactive compounds have multiple properties, including tumour preventive effects, antimicrobial, antiviral, anti-inflammatory and sedative effects [43]. The terpene content in the examined needles of fir and spruce has shown the presence of multiple terpenes that have previously been characterized by different biological activities. As in our previous research [8], spruce showed greater variability within the terpene content compared with other coniferous species, which puts this species as a possible leading coniferous tree species for further research on the impact of forests on human health. This could also contribute to the rising awareness of the importance of this species since it is expected that due to climate change its distribution will decrease [44]. Previous research confirmed the positive impact of forest therapy on human health through the increment in the number and the activity of natural killer (NK) cells known to fight cancerous cells and virus-infected cells in our body [45]. In other published research, during the first stage, the impact of certain BVOCs (terpenes called phytoncides in the specific research) was firstly examined in vitro. Essential oils from different coniferous species and specific terpenes: α-pinene, 1,8-cineole, and d-limonene were used to examine their impact on NK cells. It was concluded that these compounds significantly increased and even restored the lost activity of NK cells [46]. Further on, these findings were confirmed within in vivo experiments in the forest [47] and afterward in unnatural environments where participants were exposed to inhaling the essential oils of previously used conifer species [48]. Forest therapy sessions also showed a positive impact on the human cardiovascular system. Previous research confirmed that forest walks promote cardiovascular relaxation compared to walks in urban environments [49], reducing blood pressure and possibly preventing clinical hypertension [50] while the low levels of blood pressure remain constant even 5 days after the forest walk [51]. Spending time in forests showed positive effects on the chronic patients, showing decrement in blood sugar levels among diabetic patients [52] and decrement in chronic pain [53]. Besides the positive physiological effects of forest therapy on human health, most of the medical research in this field focused on psychosocial effects and the impact of forest therapy on different mental disorders. While investigating the impact of forest therapy on stress levels among working females in healthcare, the following findings were reported: forest therapy reduces stress levels and has relaxing effects on the parasympathetic nerves while frequent use of forest environments is more efficient than short term uses [54]. Similar results were reported while comparing the effects of just viewing forest vs. urban environments, where exposure to the forest view resulted in psychological relaxation effects on young adults [55]. While assessing the impact of forest therapy for treating the depression and anxiety among patients with chronic stroke, reported findings showed benefits of forest therapy among these patients, and it was particularly suggested that this kind of therapy could be highly useful among patients that cannot be treated with standard pharmacological or electroconvulsive therapies [56].

Terpenes are natural agents with antiviral activities that could be used to boost and/or complement standard antiviral therapy [43]. So is α-cadinol which in this investigation shown to be the most abundant compound in the examined material. Previously this sesquiterpene has been described to have antiviral properties within the research considering the search for Coronavirus natural inhibitors [24]. There are multiple paths through which these compounds act as antitumor agents in the human body [43], and recent clinical trials showed successful effects of terpenes on different human cancers [57]. In our research, most of the detected terpenes have previously been characterized to have antitumor effects, such as the most abundant compounds α-cadinol [58] and spathulenol that have also been described as immunomodulatory agents [59,60] and possible substances in the fight of drug resistance in cancer treatment [61]. Besides having antitumor effects, α-caryophyllene also showed immunosuppressive and analgesic properties [62]. caryophyllene oxide also acts as an antitumor agent and has a cytotoxic effect on different cancer cells [61], while citronellol has been shown to be an antitumor and anti-inflammatory agent [63]. The terpene screening also showed the presence of widely researched monoterpenes, α-pinene and β-pinene that have the potential to improve human health, as they act as an anti-inflammatory, antitumor, sedative, antimicrobial and antidepressant agents [17,18,64,65]. The various biological activities of detected terpenes in dominant tree species in the vicinity of investigated hiking trails give the possibility to investigate the effects of these forests on different health issues. In regard to the number of compounds that are described to have antitumor and immunomodulatory properties, the observed area could be beneficial for the development of forest-based potential health improvement programmes. Nevertheless, the presented results trigger a series of research questions and open new avenues for future research aimed at a better understanding of the causal relationships between forests and human health. 

As the research interest in the positive effects of forest therapy on human health is increasing, especially in the field of preventive medicine and public health, so does the research on forest BVOCs [8]. However, there is a huge knowledge gap considering the forest factors and characteristics, that include BVOCs, that have an influence on the outcomes of forest therapy, thus further research is highly needed in order to support the ongoing global medical research on the healing effects of forest on human health [66,67,68]. Nevertheless, this exploratory study provides a much clearer picture and more information considering forest environments for further research in the medical realm. Furthermore, it provides the departure point for the investigation of how certain environmental features and forest elements, such as dominant species could affect specific health issues, which will be particularly valuable within preventive medicine and public health [69]. Although these compounds are highly dependent on the environmental conditions, the current state of the art research in this field shows some preliminary regularities [70], which require further analysis but also set a basis for further research on BVOCs in the forest air, specifically on the role of BVOCs in forest therapy.

### 4.2. The Development of Forest Therapy Programmes Based on Health Properties of Terpenes

As discussed in the previous section, the sampling of terpenes along the hiking trails stretching through the mixed forests of beech and spruce with fir indicated their significant potential to benefit people’s health. To this end, we saw the opportunity in activating these areas for forest therapy purposes, which may consequently aid the sustainable development of NP Tara and involve the local community in unlocking the health tourism potential in the area.

The suggestion here is to offer the forest activity known as Shinrin-Yoku, translated from Japanese as forest bathing, as a way of rest, recuperation and relaxation in the arboreal environments [10,71]. All observed hiking trails can be activated for forest therapy walks, by either utilizing their full length, such as the Mitrovac—Banjska stena, or activating only some of their parts. The duration of the walks within the existing hiking trails may vary, ranging from 1 h to 5 h to cover the distance of 14.6 km of the longest trail, to the shortest one totalling 3.6 km, which provides possibilities to create different types of forest walks and adjust them to the participants. Due to the high elevation, values within certain hiking trails, such as 10 Mitrovac—Luke and 09 Mitrovac—Predov Krst limit the possibility of traversing these routes; however, their more accessible parts could be used for structured, guided walks. As the most appropriate environment for forest therapy, based on the examined features, a hiking trail 09 Mitrovac—Banjska stena was singled out (Table 1). Low values of its conditional and technical difficulty, medium elevation, and approximate duration of walk of 2 h may offer ideal conditions for forest bathing.

In profiling the forest therapy trails for forest bathing purposes, it is necessary to observe and carefully combine the different features of the trails that may influence the effectiveness and ensure easy delivery of the potential health improvement programmes. Comfort and security, alongside their health properties, are the main prerequisites for forest therapy delivery. The elevation, technical and conditional difficulty as presented in Table 1 may be limiting factors for developing forest therapy programmes. Therefore, the length and the duration of the forest walk should be in accordance with the physical preparedness of its users. In any case, however, the forest walks should not exceed 2 km in length, as previously proposed [71]. The forest bathing trail should be of mild difficulty, easy to navigate and relatively sheltered to avoid the direct emission of the sun during the summer months. 

Social scientists have explored the value and meanings that immersive and mindful forest walks hold for those who consume them [72,73]. In essence, what they found is that the close contact with the forest atmosphere reconnects people with the land and activates their senses, allowing for multisensory, affective and kinaesthetic processes. Part of these benefits come from the sensory stimuli found in forests, especially when it comes to how compounds in forest air interact with the sense of smell. It is important to mention that terpenes are responsible for the fragrance, taste, and pigment of plants [74], which is suitable for aromatherapy invitations as part of forest therapy programmes. The sensorial forest attributes, combined with the concentration of terpenes that the trees exert while emitting a spectrum of distinct scents may, therefore, additionally enhance the health benefits of spending time in forests. Some of the major benefits that have been extrapolated in previous research are embodied in the fact that these compounds have the potential to be used in therapeutic and medicinal purposes as an anti-inflammatory, antibacterial, analgesics, and many other curative, therapeutic, or preventive agents, as discussed in the previous section [74,75]. A deep inhalation of the forest atmosphere during a 2-h forest bathing walk may, therefore, be sufficient for the lungs to be filled with a variety of terpenes that have the power to address different aspects of human health [12].

Forest bathing walks are structured and led by a trained practitioner. The guides navigate the participants through slow and immersive activities which are designed to centre oneself in the present moment. During the walk, people are encouraged to stop often and sense the environment. For example, they are asked to touch the trees and plants, breathe and taste the air, and take in the sounds and sights of the forest, which all have soothing and calming benefits. The study by Farkic et al. (2021) may be useful as the authors explored the forest bathing practices in the Serbian context, suggesting what the forest bathing products may look like. The incorporation of the elements of landscape therapy with the relaxing soundscapes, spa content, such as ‘wollala’ wellness (scrubs with sheep wool), or collecting medieval medicinal herbs, may all add to the overall forest therapy experience. It would here be of particular value that the guides incorporate the information on the health aspect of forest air, by interpreting the effectiveness of terpenes in improving human health and wellbeing. 

The concept and practice of forest bathing may not only contribute to more sustainable utilisation of natural resources, but also to fostering the development of tourism activities in underdeveloped, yet pristine nature-rich destinations, such as NP Tara. The mindful utilisation of the charted forest trail network by offering forest bathing as a potential health improvement programme, the NP Tara in partnership with the local destination management organisation (DMO) can greatly contribute to the wellbeing of both forests and people. In doing so, the undertaken activities would not only support the concept of planetary health [53] but also contribute to the sustainable development of the region and ensure its long-term prosperity.

## 5. Conclusions

The examination of the terpene content in fir and spruce growing in the vicinity of the hiking trails of Tara National Park in Serbia showed the presence of compounds with a wide range of biological activities. The most dominant and abundant terpenes were α-cadinol and spathulenol which are known to act as antiviral, antitumor, antimicrobial and immunomodulatory agents. The main limitation of this study is that the content of BVOCs does not always reflect the emission of BVOCs in the air, so this research aims to give guidelines for further research that would involve medical professionals and measuring BVOCs in the air during actual therapy in order to have more reliable conclusion on the health effects of BVOCs of selected species. To this end, the environmental features of the existing hiking trails in Tara National Park provide adequate space for forest therapy programmes irrespective of age, health condition, or level of fitness. Carefully chosen trail sections with the existing walking infrastructure and qualified guiding services, alongside the vicinity of the tourism centre Mitrovac, provide a solid base for the development of a health tourism programme based on the potential health properties of tree terpenes. The defined specificity of terpenes detected in the needles of spruce and fir growing in this area could lead to the further research in preventive and public medicine and could induce more specific medical research targeting exact health issues and determining forest health effects on it, while also measuring the content of BVOCs in the air in order to conclude the possible positive effects. Further research should also aim to reconstruct the dynamics of BVOCs in the air as an advance to the current state of the art.

## Figures and Tables

**Table 1:** List of evaluated Tara National Park hiking trails with relevant features for forest therapy qualification.

Evaluated Hiking Trails	Hiking Route Length (km)	Hiking Route Elevation (m)	Hiking Route Duration (Hours)	Conditional Difficulty (out of 10)	Technical Difficulty (out of 10)
06 Mitrovac—Zborište	11.5	600	3	4	2
07 Mitrovac—Donji Jelisavčići	3.6	240	1	2	2
08 Mitrovac—Predov Krst	14.6	680	5	5	2
09 Mitrovac—Banjska stena	6	105	2	1	1
09a Mitrovac—Velika livada—Banjska stena	6.2	200	2	2	3
10 Mitrovac—Luke	12.8	780	3	3	5

**Table 2:** Major terpenes in the examined species (%). The intensity of colour is proportional to the abundance of each compound.

Compound	*Abies alba*	*Picea abies*
**Monoterpene Hydrocarbons**		
α-Pinene	1.27	0.00
β-Pinene	1.22	0.00
Camphene	0.00	0.32
D-Limonene	0.00	2.76
α-Terpinene	0.00	0.28
3-Carene	0.00	0.35
**Oxygenated Monoterpenes**		
Camphor	0.40	1.03
Borneol	13.40	0.00
α-Terpineol	0.00	1.33
Citronellol	0.00	9.22
**Sesquiterpene hydrocarbons**		
α-Cubebene	19.55	9.00
Ylangene	4.62	4.12
Copaene	1.59	0.00
α-Caryophyllene	18.92	12.23
Cedrene	3.54	0.00
α-Longipinene	0.00	12.39
**Oxygenated sesquiterpenes**		
Spathulenol	11.18	22.44
Caryophyllene oxide	9.39	15.12
α-Cadinol	25.18	9.42

## Data Availability

Data is contained within the article.
